# A critical interrogation of the legitimacy of commercial actors in food policy partnerships

**DOI:** 10.1093/heapro/daaf171

**Published:** 2025-10-07

**Authors:** Cécile Knai, Yanaina Chavez-Ugalde, Elizabeth Eastmure, Matt Egan, Harry Rutter, Laurence Blanchard, Mark Petticrew

**Affiliations:** Faculty of Public Health and Policy, London School of Hygiene & Tropical Medicine, 15-17 Tavistock Place, London WC1H 9SH, United Kingdom; The Food Policy Impact Lab, Faculty of Public Health and Policy, London School of Hygiene & Tropical Medicine, 15-17 Tavistock Place, London WC1H 9SH, United Kingdom; SPECTRUM Consortium, Usher Institute, The University of Edinburgh, 5-7 Little France Road, Edinburgh EH16 4UX, United Kingdom; Centre for Food Policy, City, St George’s University of London, Myddelton Street, London EC1R 1UB, United Kingdom; Research Office, Health New Zealand Te Whatu Ora, PO Box 793, Wellington 6140, New Zealand; Faculty of Public Health and Policy, London School of Hygiene & Tropical Medicine, 15-17 Tavistock Place, London WC1H 9SH, United Kingdom; SPECTRUM Consortium, Usher Institute, The University of Edinburgh, 5-7 Little France Road, Edinburgh EH16 4UX, United Kingdom; Department of Social & Policy Sciences, University of Bath, Claverton Down, Bath BA2 7JP, United Kingdom; Faculty of Public Health and Policy, London School of Hygiene & Tropical Medicine, 15-17 Tavistock Place, London WC1H 9SH, United Kingdom; The Food Policy Impact Lab, Faculty of Public Health and Policy, London School of Hygiene & Tropical Medicine, 15-17 Tavistock Place, London WC1H 9SH, United Kingdom; Faculty of Public Health and Policy, London School of Hygiene & Tropical Medicine, 15-17 Tavistock Place, London WC1H 9SH, United Kingdom; SPECTRUM Consortium, Usher Institute, The University of Edinburgh, 5-7 Little France Road, Edinburgh EH16 4UX, United Kingdom

**Keywords:** public-private partnerships, legitimacy, qualitative, theory

## Abstract

Public-private partnerships (PPPs) between commercial actors and governments or other non-commercial organizations are widely encouraged as a way of achieving a range of policy objectives, including the creation of healthier food environments, despite the evidence of their limited effectiveness at doing so. The aims of this qualitative study were to critically interrogate the role and legitimacy of food industry actors as partners in policies to improve the food environment, and to explore related underlying issues that impede the design and implementation of effective policies. Qualitative interviews with 16 academics from 6 countries with expertise on population food policy, including public-private partnerships, to improve the food environment were conducted from January to March 2020. A manual thematic analysis of the data was employed, and theoretical lenses relevant to the commercial determinants of health were applied. Key themes constructed from the data have been conceptualized as ‘fault lines’, metaphorically used here to indicate underlying issues or factors that cause systemic problems or impede success of public health goals. The reported fault lines are categorized as (i) uninterrogated assumptions that partnership working is effective; (ii) the role of exclusive social networks; (iii) the voluntary nature of partnerships; (iv) data ownership; (v) control of narratives; and (vi) the centrality of political ideology. This paper calls for a systematic and critical interrogation of the mechanisms and extent of commercial actors’ involvement in making decisions about healthy diets for the population.

Contribution to Health Promotion

- This study explores the influences of food industry actors on development of policies to influence food environments through public-private partnerships
- We expose significant ‘fault lines’ underpinning public-private partnerships
- Theories of corporate legitimacy, power, and policy capture are extended by accounting for how these fault lines support an ongoing assumption that public-private partnerships are the gold standard for improving the food environment.
- This study emphasises the need for continuing to analyse the uninterrogated ‘embedding’ of commercial food actors in making decisions about healthy diets for the population.

## INTRODUCTION

Unhealthy diets, especially consumption high in sodium and added sugars, and low in fruit, vegetables and whole grains, are now one of the main risk factors for deaths and disability-adjusted life years globally (GBD 2017, Diet Collaborators 2019). Unhealthy diets are significantly influenced by the activities of large multinational food and beverage corporations that are involved in the manufacture or sale of ultra-processed food and beverages, and/or high in unhealthy fats, free sugars, and/or sodium (Chavez-Ugalde *et al.* 2024, Kesaite *et al.* 2025).

Approximately ten food companies globally dominate the manufacturing, distribution and marketing of these unhealthy products (OXFAM 2014). These companies are among the so-called ‘unhealthy commodity industries’, in which a significant share of their product portfolio comprises unhealthy high-profit margin products targeted at large numbers of consumers (Knai *et al.* 2021). This paper refers to these powerful commercial actors as ‘the food industry’, acknowledging the diversity of commercial food actors in the food system. These companies have positioned themselves as strong advocates for improving food environments and diets (Knai *et al.* 2021). They do so primarily to support their business interests, and to prevent effective public health policies which are likely to become barriers to profit (OECD 2017). One of the ways in which they do so is to promote policy constructions such as partnerships between the food industry and governments and other non-commercial organizations, such as local authorities, charities and others (public-private partnerships, PPPs), to improve the food environment.

### Public-private partnerships to improve the food environment

Public health and food policy as fields of research have responded to the crisis of poor diet, with the well-documented evidence of effectiveness pointing to structural changes such as product reformulation, *trans*-fat bans and limits, front-of-pack labelling and sugar taxes (Blanchard *et al.* 2024a, 2024b). Public private partnerships (PPPs) between the food industry and governments and other non-commercial organizations have also been strongly promoted by industry and governments on the basis of the claim that they can act as population level public health interventions to improve diet- related diseases (GBD 2017 Diet Collaborators 2019). A PPP typically involves collective work between at least one private for-profit organization with at least one public (not-for-profit) organization to jointly share efforts and benefits, with a common commitment to a health outcome (Bryden *et al.* 2013). Examples of PPPs established to improve diets include the Public Health Responsibility Deal for England (Knai *et al.* 2015, 2017, 2018a, 2018b) and the Australian Food and Health Dialogue (Jones *et al.* 2016). The rationale for PPPs to improve food environments is to harness the industry’s know-how, access and established networks, offer resources, skills, supply chains and reach, making them an obvious partner in theory (Ingram and Lord 2019). For commercial partners, PPPs may provide opportunities to promote their brand and image, access privileged information that provides competitive advantage, and present themselves as legitimate actors in policy-making processes (Buse and Walt 2000, Durand *et al.* 2015, Eastmure *et al.* 2020).

PPPs to improve the food environment have been demonstrated as a failure in public policy, unlikely to result in optimal health gains (Knai *et al.* 2018a, 2018b, Blanchard et al. 2024a, 2024b). A comprehensive 2024 systematic review of the effectiveness of PPPs for improving food environments found that such PPPs are have limited if any positive effect at encouraging healthier behaviours and positively changing structural factors at population level (Blanchard *et al.* 2024a, 2024b, 2025). Indeed some studies have reported that PPPs result in estimated worsening of population health, such as a modelling study on the impact of the Public Health Responsibility Deal for England, due to lack of target setting, monitoring and enforcement (Laverty *et al.* 2019). The authors also reported a lack of cost benefit of the Public Health Responsibility Deal (Laverty *et al.* 2019). Yet in spite of the overwhelming evidence that we should be at the very least cautious about promoting PPPs as a solution for improving the food environment, they continue to be encouraged (De Pinho Campos *et al.* 2019, World Economic Forum 2023).

A central concern in health research is that governments have historically privileged economic concerns over health concerns, typically committing to ‘personal choice’ and ‘individual responsibility’ orientations of public health (Popay *et al.* 2010). In this context, partnerships with the commercial sector have become normalized. For example, partnerships with commercial actors in public health policy is regularly encouraged in high-level global health commitments, such as the United Nations Sustainable Development Goal #17 (United Nations 2025), and are variously referred to as multistakeholder alliances, intersectoral collaboration, alliances with industry, among other terms (Amri *et al.* 2022). Thus there is now an often-uninterrogated assumption by governments and across society that the food industry should be actively involved in food policy decisions. Underpinning this is an acceptance of commercial actors as legitimate leaders in food policy.

The main aim of this study was to explore the motivations for choosing, and risks and benefits of designing, implementing and managing, different types of population interventions to improve the food environment, including PPPs. Though there is now considerable evidence of how commercial actors to shape different stages of the public health policy process (Knai *et al.* 2021, Lauber *et al.* 2021), there is still insufficient clarity on how the food industry establishes itself as legitimate decision-makers in public food policies. Our analysis begins to address some of the aforementioned gaps, by advancing our understanding of corporate legitimacy through the lens of PPPs designed to improve the food environment. This study contributes to the field of research on commercial determinants of health (CDoH), defined as the ‘systems, practices, and pathways through which commercial actors drive health and equity’ which actively acknowledges the complexity of the relationship between commercial actors and health, involving political, economic, and social systems (Gilmore *et al.* 2023). Of particular relevance to this research are the well-documented ways in which commercial actors have successfully promoted one of their preferred policy designs, PPPs (Mialon *et al.* 2015). There are several theoretical lenses through which to understand the CDoH, and specifically the research reported here. In particular we draw on theories of corporate legitimacy, power, and policy capture.

### Theoretical foundation

Legitimacy is conceptually presented both as a normative phenomenon (what *should* be legitimate forms of authority) (Buchanan and Keohane 2006), and as a sociological phenomenon, where legitimacy is an outcome of beliefs about an actor’s right to make decisions and to have authority over an issue (Bernstein 2011). In her study of global partnerships for better nutrition, Lie (2021) posits that both conceptualizations of legitimacy are relevant as they are shaped by norms about the exercise of power, which are in turn reflective of societal beliefs (Lie 2021). Dowling and Pfeffer’s Theory of organizational legitimacy (Dowling and Pfeffer 1975), also referred to as Legitimacy Theory (Clapp and Fuchs 2009, Velte 2023) sets a highly relevant foundation for which organizations including commercial actors deploy ‘legitimation behaviours’ to establish societal acceptance, including aligning with political leaders, making charitable contributions and engaging with sustainability and environmental goals (Velte 2023). A recently introduced Theory of Elite Influence and Popular Legitimacy draws from research on citizen and elite attitudes towards international organizations (Dellmuth and Tallberg 2023). We will refer to this work particularly with regard to commercial actors employing instruments in their position of power to garner credibility among citizens and to shape the narratives and citizens’ political beliefs (Dellmuth and Tallberg 2023). Commercial actors seek to legitimize themselves through a range of approaches, including discursive strategies, communicated through language and the use of argument and reasoning about why a partnership is legitimate, e.g. invoking successful past collaborations and deeply entrenched community roots (Gronau and Schmidtke 2016, Blanchard *et al.* 2024a, 2024b).

Interlinked with the concept of legitimacy is that of power, variously defined as ‘the production, in and through social relations, of effects that shape the capacities of actors to determine their circumstances and fate’ (Barnett and Duvall 2004) or ‘capability’ or ‘transformative capacity’ (Gaventa 2006). Theories of power, notably Lukes’ three dimensions of power (Lukes 1974), Barnett & Duvall typology of power (Barnett and Duvall 2004), Gaventa’s ‘power cube’ (Gaventa 2006) and Gramsci’s theory of hegemony (Bates 1975) can help explain phenomena such as corporate legitimacy by delving into the many facets and applications of power. These include notions ranging from overt, direct action to control or coerce (e.g. gaining institutional or structural advantage), to shaping of societal norms, beliefs and ideologies, (e.g. through processes such as framing, narratives and knowledge-production to influence the way people think, believe and act).

Policy or regulatory capture, the process of systematically ‘directing public policy decisions away from the public interest towards the interests of a specific interest group or person’ (OECD 2017), is useful as a theoretical framework as it sheds light on how commercial interests unduly influence public institutions and policies to service business interests. The theory originates in the field of economics, most notably George Stigler’s Theory of Regulation (Stigler 1971, Carrigan and Coglianese 2016).

## MATERIALS AND METHODS

### Study design

This study aimed to understand the motivations for choosing, and risks and benefits of designing, implementing and managing, different types of population interventions (including PPPs) to improve the food environment, and to advance our understanding of corporate legitimacy. We conducted a qualitative interview study with international academics with expertise on population food policy, including PPPs. These interviews helped inform an authoritative systematic review on the effectiveness of different types (mandatory, voluntary, or partnerships) of population interventions to improve the food environment (Blanchard *et al.* 2024a, 2024b).

### Sampling and recruitment

We aimed to interview stakeholders with the unique academic or policy expertise and experience in designing, implementing, and/or evaluating population interventions to improve diet, with experience of evaluating or otherwise studying PPPs. Commercial actors were not interviewed (Thomas *et al.* 2024). Key stakeholders were determined from preliminary, desk-based research from relevant literature, and in consultation with collaborators working on relevant topics. Candidate interviewees from relevant organizations were identified online and approached via e-mail with an information sheet about the study.

### Data collection

The interviews were conducted online by a social epidemiologist (YCU) with experience in qualitative research methods, in February and March 2020. All interviews followed a similar structure and topic guide prepared by the principal investigator, namely to explore: any current and recent population interventions to improve diet and their evaluations; types of interventions i.e. mandatory, voluntary, PPPs; their understanding of why certain types of interventions might be selected by those designing and/or implementing them, including motivating factors (such as scientific evidence, political expediency, etc.), benefits and risks. Interviews were audio-recorded with participant consent. They were then sent for transcription to a transcription company (Way with Words) via their further encrypted transcript uploading system. The transcriptions were returned de-identified for analysis.

### Data interpretation

A reflexive thematic analysis was employed to construct meaning from the interview data (Braun and Clarke 2006, 2021). Our theoretical lenses informed the analytic process. Specifically, the analysis was primarily deductive, guided by established theoretical constructs that sensitized the researcher to patterns in the data related to how corporate actors seek to legitimize their influence, exert power across systems, and capture policy-making processes. For example, codes reflected manifestations of discursive legitimacy and regulatory influence. The analysis also allowed for inductive insights where participants offered unanticipated perspectives.

The analysis and manuscript drafting processes were disrupted by the COVID-19 pandemic, and overshadowed by unavoidable immediate priorities, and so planned collaborative analysis and writing was conducted in stages. Thus, an initial set of codes was deductively generated by one co-author after carefully reading through the transcripts, and shaped by existing concepts as reflected in our topic guide. A second round of coding was conducted by another co-author. The final set of codes was shared and discussed with the project lead, and the final coding frame was used to code all interviews: one co-author prepared a first complete thematic presentation of the data as charted in an Excel spreadsheet matrix, and after discussion with the project lead, a draft of the results was prepared. Finally, the project lead expanded on the initial analyses and drafts, conducting an exploration of the data focusing mainly on PPPs, but also providing an account of the complexities and nuances by reporting examples of where public health policy makers managed to influence decisions on voluntary approaches despite the pressure for partnering with the industry.

### Positionality

As academics in the field of food policy and the CDoH, we acknowledge our extensive experience conducting research on these topics and thus we do not come to this topic blindly. As such, we are committed to the production of unbiased and methodologically robust scientific knowledge production, with a view to improving public health.

### Ethical considerations

Ethical approval was granted by the London School of Hygiene & Tropical Medicine’s Observational Research Ethics Committee (Ref: 19118).

## RESULTS

### Participants

Interviews were conducted with 16 experts based in six countries: Australia (3), Brazil (1), Canada (3), France (1), the United Kingdom (5), and United States (3). Given the relatively small community of experts on this topic, and in accordance with the study design, we are not providing further participant identifiers other than the country in which they are based.

We organized our findings by the key themes constructed from data. The themes have been conceptualized as ‘fault lines’, metaphorically used here to indicate underlying issues or factors that cause systemic problems or impede success of public health goals. The reported fault lines of partnerships with the food industry are thus categorized as (i) uninterrogated assumptions that partnership working is best; (ii) the role of exclusive social networks; (iii) the voluntary nature of partnerships; (iv) data ownership; (v) control of the narrative; and (vi) the centrality of political ideology.

### Uninterrogated assumptions of partnerships as the ‘gold standard’

Several interviewees reported that there is a general reluctance among health policy makers to ‘*work against’* industry. One interviewee recalled the assumption that the food industry would not be regulated, in the process of negotiating one of the most influential and politically fraught World Health Organization global strategies, notably the 2004 WHO Global Strategy on Diet, Physical Activity and Health (World Health Organization 2004):

[…] the WHO global strategy on diet 2004, I think […] was very much talking about all of the stakeholders, governments who work with the industry, […] so it was very strategic in terms of defining the type of approach that public health and ministries of health should take. […] Everything was really about [industry] self-regulation […]. (P1)

Interviewees suggested the starting position for development of food policy often included an assumption that policy would be developed in partnership with industry groups, consistent with industry positioning themselves as part of the solution. Assumptions about partnership working were also described as often limiting the policy options available to officials to voluntary agreements or supporting industry derived self-regulation. Interviewees explained the resulting impact on policy making, characterizing the situation as more often than not excluding more stringent mandatory or regulatory approaches. For example:

[…] people I've talked to in the government and people who were involved in the [policy] development told me that right from the beginning the question of having a mandatory system… having the most stringent system was not even discussed. It was said that the government should be working with the industry. When everyone was around the table this was [the] starting point for everyone. (P1)

The limited effectiveness of partnership working to improve the food environment was acknowledged, with one interviewee noting that

[…] in the last five years we are starting to realise that it's not working and we are going nowhere with that. [However] for the food industry, the big partnerships have been the ‘gold standard’ for many years. (P1)

Some interviewees argued that there is a role for industry at specific stages of the policy process:

Of course, people that are representing industries that have vested interests need to be partners in implementation. Where I think things go very awry is where they’ve got, they, they sit at the table about policy formulation or development programmes. So I agree with the WHO view that those with vested interests, particularly commercial interests, should not be part of policy development.[…] But after the policy is developed, sure, there's a role. The food industry hate that sort of language here in Australia. They say they're part of the solution and that we're excluding them from being good corporate citizens by those views, etcetera. And I don’t, I'm not fooled by that. (P7)

Reported potential reasons for health decision makers to default to a partnership approach included the political power of industry, expressed in terms of employment and other economic arguments, and financial contributions to political parties, as explained here:

understanding context is important, so both the context of what's needed in terms of the food system, better diets, or the health system or whichever system you're looking at. Um, but also the political context, you know, who’s got the power and where’s the consolidation of power in that particular country or food system, um, and therefore whether your, you know, perfect technical intervention in to X, Y or Z, that, that public health people have designed, whether it's even gonna fly because of the, the political nature of policymaking and, um, and how things get done. (P4)

### Exclusive social networks: ‘they often…go to the same schools’

Interviewees reported how commercial actors exploit the principle of reciprocity by intentionally cultivating relationships with policy makers, as noted by two experts:

They trade on the reciprocal nature of human nature relationships. And they know what they’re doing. (P10)

So, I know that […] they're knocking on doors, they've got very close relationships with the ministers, and they can also call on the huge financial contribution that they provide to the country and that this will impact on sales. (P14)

Personal relationships between political, government and commercial actors, based on friendships and socializing together, and relatedly, access to privileged positions of power, were raised as an important factor facilitating a partnership approach:

what I've seen in many countries is that policymakers they are from a certain class. […] From the elite and they often grow up in the same cities and go to the same schools with lobbyists, with people then managers or CEO of companies. […] So when it comes to taking decisions they will not take decisions against […] the system because […] ‘we want to work with everyone, everyone has the freedom to say what you eat’. But also because ‘he is my friend and tomorrow I will play golf with him’. (P1)

Such past and present relationships were reported to create incentives to carry on regardless of whether the partnership was meeting targets or not, as explained by an interviewee:

[…] because the government is so much linked with the industry and these types of projects, the government will never officially say, oh we failed, it wasn't a good idea after all. (P1)

### The voluntary nature of partnerships: ‘based on what the industry wants’

A primary concern with voluntary agreements was about setting of targets and commitments, with industry groups often committing to targets they were expecting to achieve or had already achieved prior to enacting the agreement. As explained by one interviewee,

The public health groups said they knew [the industry targets were] going to happen anyway, they [the industry partners] were just following what their forecasted trends were.’ (P6) […] [One of] the largest food retailer in the world […] made commitments about reducing sodium, sugar, fat, trans fats… [but …] they actually had achieved their targets for those nutrients of concern before they even made the announcement. (P6)

Industry involvement at the design stage of agreements was reported of relevance to the voluntary nature of the intervention, as industry perspectives were given considerable weight. Moreover, interviewees explained that commercial perspectives at the design phase of policy was considered crucial to ensure that commercial actors would be more likely to sign up. As recalled by an interviewee,

As it was voluntary, the way that the government led the process was based on what the industry wanted. Because they [the industry] were saying, ‘it’s voluntary, we are going to do what we can do’… they have their arguments on technology and capacities and reformulation issues [… Yet] we don’t have […] strong bodies to build voluntary agreements with […] balancing the forces. (P9)

The lack of a countervailing or balancing view in the setting of the targets was often an important weakness in the design of the voluntary agreement. The need for a balancing party presents a dilemma for the public health community as the public health researchers we interviewed highlighted a reluctance and potential conflict of interest inherent in working with industry groups, yet voluntary agreements that are developed in the absence of the public health voice are considered weak. Relatedly, concerns about the overall lack of transparency in partnership agreements were noted, e.g.:

[…] the way that the voluntary agreements were developed, there was no transparency in the process… […] and the targets are ridiculous. (P9)

Interviewees discussed challenges with monitoring and enforcing interventions, including the allocation of sufficient funding and the capacity to undertake monitoring activity. One interviewee suggested that while the focus was on development of new policy interventions the lack of funding for monitoring prevented basic requirements, e.g. review of dietary guidelines, to be met.

One of the problems that I have is when we look at population interventions to improve diet, a lot of the… sexy policy actions, like labelling, or you know, reformulation get a lot of focus, but our basic tools that we need for evidence informing nutrition policy, such as data from regular coordinated food and nutrition monitoring and surveillance systems, or tools that we get from national appropriate food based dietary guidelines, aren't on the list. (P7)

While difficulties with undertaking monitoring were highlighted, some interviewees described examples of effective monitoring. For example, an interviewee described implementation of monitoring of food marketing, where the monitoring framework had been developed to support new legislation. While the legislation was not approved in the final stages, funding had been allocated to implementation of the monitoring framework, and monitoring was put in place to determine baseline activity across a range of media, in the expectation that the legislation was imminent. Interviewees also described non-governmental organizations participating in monitoring, e.g. registering instances of activity that were not compliant with regulations on a website, to supplement limited government monitoring and enforcement activity, and consistently reporting violations of marketing regulations.

### Data ownership: ‘he industry possesses the data. We don’t have access’

Linked to the voluntary nature of partnerships is the issue of data ownership. As suggested by one interviewee, the government as a partner was inevitably vested in the partnership’s success and less able to direct or control the nature of the commitments.

Companies pretty much decide what they want to do. They have the most up to date information… and you know they can put in what they want because the government really doesn't have the data. (P1)

Interviewees explained that this had a direct impact on the government’s need to maintain credibility and the partnership not being seen to have failed:

[Partnerships are] also a risk for the credibility of the governments because […] when […] the partnership fails, you can't [say] that because you will be saying that you wasted time, you wasted money from the taxpayer. So you will always have to find some benefits to what you did even if it's not true. […] You [the government] are […] tangled in this partnership in a certain way […] unless there is independent evaluation which is difficult to make because […] the industry possesses the data. We don't have access. Very often we don't have access to the data. (P1)

Access to the full data was considered all the more important in light of the knowledge that evidence is but one component of decision-making around public health policy, and that some policy was made in the absence of evidence. Thus, being armed with the full data to conduct an evaluation which reflected the reality of a partnership was considered crucial:

[Evidence] is important to have, but there’s many other things that are perceived to be more important by decision makers… whether they can sell it to their constituents… (P7)

Interviewees identified the need for public health researchers to be prepared with the best data so that they were able to act when these policy opportunities presented themselves:

I think the onus on the scientists and the policy makers, so people in regulatory bodies, is to work together to come up with the options and have them in the back pocket, like always be ready. Have the evidence, have the ideas all ready to go. And be paying attention to when… the windows of opportunities are open… that's the best we can do. (P15)

### Control of the narrative: ‘they may talk public health, but…’

Interviewees noted that public opinion and political will were linked, and that key stakeholders, including food industry representatives, were able to influence both aspects of political decision-making, e.g. with use of framing devices such as ‘nanny state’ and calling on protection of individual freedoms. The use of ‘nanny state’ was thought to be especially difficult to counter, and interviewees suggested public health researchers needed to be thoughtful about how to create counter-narratives to such a negative characterization of government. This viewed as especially important, because communicating the benefits of nutrition to individuals is difficult, whereas restrictions on individual autonomy were more easily conveyed. Several interviewees viewed the absence of countervailing health-focused voices and viewpoints as a major problem, in the light of potential conflict of interest when working with commercial actors. As reported by an interviewee:

They have their arguments on technology and capacities and reformulation issues… we don’t have… strong bodies to […] balance the forces. (P10)

Many interviewees were opposed to the involvement of industry in policy development, with interviewees noting the conflict between the processed foods industry profit-making objectives and public health initiatives, e.g.:

Whenever they have a role, whenever they’re in the room, whenever they have a place at the table, they may talk public health, but their agenda, their reporting mechanisms will be to maximise profit for shareholders. (P10)

One interviewee suggested industry should be involved in policy implementation.

People that are representing industries that have vested interests need to be partners in implementation. Where I think things go very awry is where… they sit at the table about policy formulation or development programmes. (P7)

Moreover, interviewees noted that industry may not necessarily be opposed to regulatory approaches, and that factions within industry sectors may even welcome it, e.g. when companies may wish to focus on health but are reluctant to lose a competitive advantage (a so-called ‘level playing field’, requiring that all businesses comply with the same rules.

You should not make assumptions about behaviour of different players, you should go and check it out… with the salt reduction work in the 2000s […] there was an assumption that there would be strong push back all across industry. But […] distributors and supermarkets […] did their sums, and they said yeah we can live with this. So suddenly they were neutralised, or even on-side, and then it was just the manufacturers. (P10)

While regulatory approaches were preferred over other policy interventions for managing public health impacts, interviewees noted that they were not without flaws. For example, they noted that the development of well-designed regulation was challenging, and that regulation did not prevent companies from making health claims that competed with the intent of the labelling regulations:

One of the things we are realising […] is that […] regulation didn't prevent companies from also making […] nutritional claims, functional claims, those sort of things on the packaging […] and as the consumer you see, oh! this is high in sugar, oh! but it's also high in Vitamin C… A stronger policy… would be any products that has any of these warning labels shouldn't have any claims… Don't confuse the message, it needs to be a very simple message. (P15)

Thus well-crafted regulation and a vigilant and engaged public health community were considered all the more important because of the relentless efforts of the industry to protect its interests. By way of illustration, one interviewee drew on experience with the tobacco industry to explain how dynamic and fast moving commercial actors are:

[…] what we’ve learnt with the tobacco industry is they never give up, ever. They fight every inch, and even when you think that the war’s over it’s never over, they will come back. So electronic cigarettes is a great example of people going round the world saying ‘hey we’ve won the tobacco control argument’. And then suddenly, […] you have tobacco industry people popping up in ministries of health saying we’re part of the solution, we’re not part of the problem. And winning some of the public health people on to their side, getting responsibility, and normalising smoking process again. Getting people saying nicotine addiction in teenagers is fine, it’s a small price to pay for quitting in adults… ah, bizarre things. So […] you think they’re dead in the water. They’re never dead, they’re just pretending. (P10)

### Political ideology: ‘Legislate as little as possible’

Interviewees identified political ideology as a fundamental driver for the adoption of partnership approaches. As explained by an interviewee:

I think what's really important is to understand political ideology as well… a lot of people, I think, in our sector they don't understand that it doesn't matter how much evidence you put forward, if there's a political party in charge who's ideology is… to legislate as little as possible, they will not legislate on certain issues, and voluntary is the only option available to them. (P14)

Interviewees noted that public opinion and political will were linked, and that key stakeholders, including food industry representatives, were able to influence both of these aspects of political decision-making, e.g. with use of framing devices such as ‘nanny state’ and calling for protection of individual freedoms. As further elaborated by an interviewee:

Beliefs, values, ideology, I think, are the key drivers. What would maintain votes? What would keep votes? What doesn't lose votes? […] I feel like we're quite naive in health, where we think: ‘If I could just get enough evidence and then things will change. Or, if I could just present it in the right way and then things will change’. (P14)

## DISCUSSION

Our interviews with 16 experts across six countries highlight significant ‘fault lines’ or underlying systemic issues impeding the success of partnerships between the public and commercial sectors in efforts to improve the food environment.

By exposing some of the significant fault lines of PPPs to improve food environments, this study extends the theories of the CDoH by illustrating the complex ways in which acceptance of PPPs as a purported gold standard for improving the food environment has become embedded in public debates, despite the documented evidence of overall ineffectiveness.

The first reported fault lines in partnerships with the food industry relate to uninterrogated assumptions that partnerships represent the ‘gold standard’. This finding is alluded to in other studies and reports (Bruno and Karliner 2000, Moodie *et al.* 2013). In their study on how ultra-processed food industry actors attempted to influence non-communicable disease policy at the World Health Organization, Lauber *et al* (2021) report interviewees’ experience of continued food industry opposition to regulatory approaches in favour of voluntary or partnership measures (Lauber *et al.* 2021).

In many ways, this finding (of partnerships as the ‘gold standard’) can at least partially be explained by the five other themes in the research. In other words the legitimacy of partnerships with the food industry as the presumed best way forward to improve the food environment is reinforced by factors such as elite networks and shared political ideology of commercial partners, as well as the nature and functioning of the partnership itself.

There is a large body of research on elite social networks and how they perpetuate positions of power, influence and legitimacy, both internally and across public opinion (Santoro *et al.* 2021, Li 2023). As elucidated by Gramsci in his theory of hegemony, the elite class maintains power not only through direct influence but also through ideological dominance, by shaping belief systems, including political ideology, that govern society (Bates 1975, Sociology Institute 2022). Political ideology and specifically the belief that minimal legislation is the best way forward, was identified as one of the ‘fault lines’ constructed from the qualitative data. Differences in political ideology are of course acceptable and important, nevertheless here political ideology is mentioned by interviewees as an instrument with which to wield power. Theories of power help to explain this phenomenon further, with e.g. Steven Lukes’ ‘third dimension’ of power exploring how norms and ideas, including political ideology, can be employed to deliberately influence belief systems and behaviours (Lukes 1974). John Gaventa refers to ‘invisible power’ wherein the thinking and behaviours of an elite can pervasively influence societal norms (Gaventa 2006). Commercial actors effectively use these strategies to promote partnership working as a preferred policy approach, employing invisible, ideological power to shift norms at a systems level (Knai *et al.* 2018a, 2018b). A study on the policy process of front-of-pack labelling in Colombia cited hindering factors such as the industry deployment of legal threats, lobbying government, and engaging high ranking public officials. Competing ideologies and the ‘market-centric’ logic of government decision making were also cited as factors which negatively affected restrictions on marketing of unhealthy foods to children (Mialon *et al.* 2020). Data ownership by commercial partners was highlighted by the interviewees as a challenge in partnership working. This is likely a form of power, where a voluntary participation protects partners from sharing data and crucially from reporting on compliance, and/or a government reduces the burden of compliance for industry to maximize participation in the voluntary agreement (Bryden *et al.* 2013). A national salt reduction intervention in Fiji included voluntary engagement of the food industry to adhere to salt reduction targets; the strategy to engage industry actors was unclear, with no compliance mechanisms in place (Webster *et al.* 2018). Evaluations of partnership working in Australia (Elliott *et al.* 2014, Jones *et al.* 2016) and in England had similar findings. For example the Public Health Responsibility Deal for England evaluation reported that they shared little data, despite the expectations to do so as a partnership driven by the government; and what did they did share added little to understanding the added value of the PPP as most of the actions they undertook were already underway (Knai *et al.* 2015, Knai *et al.* 2017).

One of the ways in which ideological dominance is established and maintained is through framing an issue in a certain way, and employing certain narratives, to affect people’s understanding and feelings about the issue and control the agenda (Maani *et al.* 2022), as highlighted in the interviews. All institutions develop legitimation narratives, to emphasize a selective set of reasonings about their authority (Tallberg and Zürn 2019). Commercial actors are no different, deploying narratives about the promotion of partnership as a preferred and effective policy construct despite its ineffectiveness. (Castronuovo *et al.* 2017, Brandon *et al.* 2020, Blanchard *et al.* 2024a, 2024b). An analysis of partnership working in food policy in Australia between 2007 and 2018 reports the food industry’s use of ‘leadership’ frames such as ‘choice giver’, ‘role model’, ‘master negotiators’ (Brandon *et al.* 2020). Campbell *et al* (2020)’s framing analysis of the industry lobby against the sugar tax in Ireland also found that the food industry emphasized ‘progress’ made on voluntary actions without specifics and positioned themselves as ‘industry leaders’; demanding a seat at the policy making table, appealing to historical roots and reminding how they are firmly established in, and central to, the fabric of society (Campbell *et al.* 2020). A process evaluation of the sugar-sweetened beverage tax in France found that food industry firmly and publicly opposed the tax, but then became open to contribute to resolving the public deficit, provided the tax rationale is not public health oriented, positioning themselves as ‘concerned role-models’ within the community (Le Bodo *et al.* 2019). These examples from the recent literature are consistent with the interview findings and further our understanding of the legitimation of industry as self-proclaimed leaders in food, as they ‘assume the role of the choice giver’. This is a positive, confident act, assuming the role of making a decision about what a consumer should do or not (Blanchard *et al.* 2024a, 2024b). The deployment of narratives is also an act of creating diverse alliances and a multiplicity of connections.

The dominant place of the food industry in food policy and decision making, as reflected by expert interviews, and equally strongly reflected in policy process studies cited in this paper, raises a central question of what legitimacy, and indeed technical competence, the food industry has in designing and implementing public health policies to improve the food environment. When commercial actors are able to dominate the narrative and set the agenda of public discourse, they acquire a disproportionately high ability to ‘define’ a public process and thus gain legitimacy in ways that are not beneficial for public health (Lie 2021). Capture theory sheds light on how actively favouring partnerships as the preferred policy construction is a documented impact of policy capture by the industry (Miller and Harkins 2010), among several other documented damaging impacts of policy capture: these include misallocation of resources to accommodate special interests and reflect the interests of elites (OECD 2017), and ‘regulatory chill’, referring to abandonment, inertia or delays in policy processes (Schram *et al.* 2018, Tienhaara 2018, Hawkins and McCambridge 2021). Finally corporate legitimacy theory (Dellmuth and Tallberg 2023) can help take notice of the subtle changes over time of the conceptualization of ‘leadership’ in food policy, and structural embedding of commercial actors in public policy.

### Limitations

Participants are mostly from high income countries and a broader representation of experts from several regions of the world is missing. At the proposal stage of our study, there were very few real-world evaluations of PPPs available; we now know, having completed the systematic review (Blanchard *et al.* 2024a, 2024b), that of the nearly 500 studies evaluated, 81% of publications focused on only 12 countries (USA, UK, Australia, Canada, Mexico, Brazil, Chile, France, Spain, Denmark, New Zealand, and South Africa). The lack of perspectives from a far wider net of countries and regions will inevitably have affected the results and what the literature suggests about public private partnerships in these countries, and thus addressing inequalities in available studies is a crucial next step.

## CONCLUSIONS

This work sits in a growing body of research increasingly clarifying the role and interests of commercial actors in food policy. This qualitative study exposed significant ‘fault lines’ or underlying systemic issues impeding the success of partnerships between the public and commercial sectors in efforts to improve the food environment. Applying theories of corporate legitimacy, power, policy capture has helped explain not only the fault lines such as deployment political ideology and control of data and the narrative, but also how they are tightly interwoven to support an ongoing assumption that PPPs are the gold standard for improving the food environment. This paper demonstrates that although we are gaining clear insight into the strategies and practices of commercial actors in policies to improve the food environment, via a growing literature on the CDoH and on the policy process, there is a need for greater critical analysis of how commercial actors become de facto legitimate decision makers in food policy.

## Data Availability

The data underlying this article cannot be shared publicly to protect the privacy of individuals that participated in the study.
